# Patient Characteristics and Clinical Outcomes with Low-Dose Dabigatran

**DOI:** 10.3389/fcvm.2017.00042

**Published:** 2017-07-10

**Authors:** Ramin Ebrahimi, Janet K. Han, Seung H. Goe, Michelle Treadwell, Zenaida Feliciano

**Affiliations:** ^1^Department of Cardiology, Veterans Affairs Greater Los Angeles Healthcare System, Los Angeles, CA, United States; ^2^Department of Medicine, University of California, Los Angeles, Los Angeles, CA, United States; ^3^Department of Pharmacy, Veterans Affairs Greater Los Angeles Healthcare System, Los Angeles, CA, United States

**Keywords:** low-dose dabigatran, atrial fibrillation, dabigatran 75 mg, oral anticoagulant, patient demographics

## Abstract

**Background/aims:**

Dabigatran, an oral direct thrombin inhibitor, is used in patients with non-valvular atrial fibrillation to reduce thromboembolic events. Whereas the 150 mg dosing regimen has been extensively studied in clinical setting, to date, there is no clinical data on the 75 mg (low dose, “LD”) regimen. In this study, we evaluated patient characteristics and clinical outcomes in 49 patients treated with LD dabigatran.

**Methods:**

Electronic medical records were utilized to compare patients from one medical center treated with LD dabigatran to those from the warfarin arm of the RE-LY trial.

**Results:**

Compared to those from the warfarin arm of the RE-LY trial, the LD dabigatran patients were significantly older (82.6 vs. 71.6 years, *p* < 0.001), had higher prevalence of diabetes (42.9 vs. 23%, *p* < 0.001), were predominantly male (100 vs. 63.3%, *p* < 0.001), and had higher CHADS2 score (2.8 vs. 2.1, *p* < 0.001). Only 9 (18%) patients had creatinine clearance of <30 ml/min and none were on concomitant medications that required dose adjustment to LD dabigatran. During a mean follow up of 10.1 months, there were no thromboembolic events, no cerebrovascular events, and seven bleeding events in the LD dabigatran group of which only two required blood transfusion.

**Conclusion:**

In this database, most patients received LD dabigatran based on characteristics not related to the approved indications for this dose. The exploratory clinical outcomes of using LD dabigatran outside of the current approved indications are promising in this high-risk population and deserve further investigation to better understand the role of LD dabigatran in clinical practice.

## Introduction

Atrial fibrillation (AF) is the most common arrhythmia in clinical practice (1). Approximately 2.7–6.1 million people in America have AF (2). Prevalence of AF is 0.4–1% in general adult population (1, 3) with an increasing incidence with increasing age. In patients over the age of 80 years, the prevalence may reach as high as 8–10% (4–6). It is estimated that by 2050 as many as 12 million people in United States (US) may have AF (7). Because of increased risk of thromboembolic events, anticoagulation remains a crucial treatment plan for the majority of patients with AF including those with non-valvular AF (NVAF) (5). For approximately 50 years, warfarin served as the only oral anticoagulant available for stroke and systemic embolism prevention in AF (8–12), with on-treatment analyses showing greater than 80% efficacy in preventing thromboembolic sequela. However, the risk of bleeding is increased in such patients, especially with increased age, comorbidities, and intensity of anticoagulation (13, 14). Given its narrow therapeutic index and significant food and drug interactions, warfarin use has proven to be complicated requiring frequent blood draws and challenges keeping anticoagulation within goal.

Recently, direct oral anticoagulants (DOACs) have been studied as alternatives to warfarin in NVAF (15–18). In the RE-LY trial, dabigatran given 150 mg twice daily (BID) was found to be superior to warfarin for reduction of thromboembolic events in patients with NVAF while providing similar safety outcomes (15). The 150 mg dosing was approved by food and drug administration (FDA) for use in patients with NVAF to reduce stroke and systemic embolism. However, the populations in these phase 3 trials were quite different than what has been seen in clinical practice, where patients tend to be older and have more comorbidities, including most importantly, renal dysfunction (19). Early after FDA approval of dabigatran in 2011, there was a notable uptick in reports of serious bleeding and thromboembolic events especially in the elderly (20, 21). Although a 75 mg dose (low dose, “LD”) was also approved for patients with significant impairment of renal function, as well as those concomitantly treated with drugs that interfere with the elimination of dabigatran (cytochrome p450 inhibitors and P-glycoprotein inhibitors), this lower dose was approved solely based on pharmacokinetic data. Lack of reversibility has also been a limiting factor for utilization of DOACs in general. Recently, idarucizumab was approved for reversal of dabigatran (22), reigniting interest in the use of dabigatran, including LD dabigatran. In addition, despite development of a clinical assay to evaluate dabigatran levels, when taking into consideration accessibility of attaining these specialized laboratory test and taking into account patient preference to avoid such testing in the first place, it may be more practical to use lower doses if they are found to be both safe and effective (23).

Lack of clinical data with the use of low-dose dabigatran continues to remain a concern among clinicians interested in using this dose (24). In this study, we compare patient characteristics for those treated with LD dabigatran from one medical center to subjects treated with warfarin from the RE-LY trial. We also present exploratory data on safety and efficacy of LD dabigatran. To our knowledge, no such clinical data on LD dabigatran have ever been published.

## Materials and Methods

This is a retrospective single-center study performed at a Veterans Administration Hospital in the United States. Study protocol was reviewed and approved by the local Investigational Review Board. Electronic medical records and pharmacy database from the Veterans Affairs Greater Los Angeles Healthcare System were utilized to acquire all data for patients on LD dabigatran, and all data were de-identified to assure patient confidentiality All patients treated with 75 mg BID dosing of dabigatran between January 1, 2011 and June 30, 2014 were included in the study. Follow up data were collected until June 30, 2015. There were no exclusion criteria. A systematic review of each patient’s electronic medical record was performed by a pharmacist and a cardiologist. Patient data, including demographics, comorbidities, duration of treatment, and clinical outcomes such as bleeding and thromboembolic events were collected. The primary goal of our study was to understand the characteristics of patients treated with LD dabigatran and their comparison with those from the warfarin arm of the RE-LY trial. Additionally, we present exploratory clinical data on thromboembolic and bleeding events with the LD dabigatran population. Thromboembolic and major bleeding events were defined based on the RE-LY trial.

Review of all data was independently performed by one physician and one pharmacist. The primary investigator made the final decision in case of any discrepancy or disagreement between the pharmacist and the physician. Summary statistics (means, SD, medians, interquartile ranges, minimum and maximum values, and frequency distribution) were generated for patients on low-dose dabigatran. Chi-square test or Fisher’s exact test were used to compare categorical variables between subjects on 75 mg BID vs. the values from the warfarin arm of the RE-LY trial and *t*-test or Wilcoxon rank sum test was used to compare continuous variables. As the number of clinical outcomes such as thromboembolic or bleeding events were too few in the LD dabigatran to allow any statistical analyses, the incidence of these outcomes were only reported without any comparison. By definition, only *p*-values of <0.01 were considered to be significant.

## Results

The demographics and conventional clinical characteristics of our patients on LD dabigatran 75 mg BID and the warfarin arm from the RE-LY trial are presented in Table 1. The patients on LD dabigatran were significantly older (82.6 vs. 71.6 years, *p* < 0.001), had higher prevalence of diabetes (42.9 vs. 23%, *p* < 0.001), were predominantly male (100 vs. 63.3%, *p* < 0.001), and had higher CHADS2 score (2.8 vs. 2.1 *p* < 0.001). The mean CHA2DS2-VASC score for patients on low-dose dabigatran was 4.2. Only 9 (18%) patients had creatinine clearance (CrCl) of <30 ml/min and none were on concomitant medications that would require dabigatran dose adjustment. In the LD dabigatran population, there were a total of seven bleeding events including three gastrointestinal, two genitourinary, one hematoma, and one subconjunctival bleeding. Of the gastrointestinal bleeds, one was assessed to be from an upper gastrointestinal source and two were of lower gastrointestinal source, specifically rectal. Two patients, both with gastrointestinal bleeding, received red blood cell transfusion. There were no thromboembolic events or intracranial bleeds in the low-dose dabigatran group.

**Table 1:** Baseline characteristics.

	RELY warfarin population (*n* = 6,022)	Dabigatran 75 mg (*n* = 49)	*p-*Value
Duration of Rx (months)	24	10.1	<0.001
Age (years)	71.6	82.6	<0.001
Male	3,809 (63.3%)	49 (100%)	<0.001
Creatinine clearance (ml/min)	Not available	37.7	
DM	1,410 (23.4%)	21 (42.9%)	0.001
HTN	4,750 (78.9%)	41 (83.7%)	0.52
LVEF	Not available	51.5	
Hx CAD	Not available	10	
Hx stroke/TIA	1,195 (19.8%)	4 (8%)	0.046
Hx bleeding	Not available	11 (22.3%)	
CHADS2	2.1	2.8	<0.001

## Discussion

Dabigatran, an oral direct thrombin inhibitor has been in the US market for over 5 years. As of the date of submission of this manuscript, there still exist no US data on patient characteristics or clinical outcomes such as thromboembolic or bleeding events for patients on dabigatran 75 mg BID. Furthermore, the subjects in these phase 3 trials were quite different than those seen in clinical practice, where patients tend to be older and have more comorbidities, including renal dysfunction (19). In addition, early after FDA approval of dabigatran in 2011, there was a notable uptick in reports of serious bleeding and thromboembolic events especially in the elderly (20, 21). The above findings have contributed to limited use of the LD dabigatran and serve as the premise based on which the current project was performed. In this study, we presented patient characteristics of those treated with LD dabigatran and compare them to those from the warfarin arm of the RE-LY trial. We also presented exploratory data on bleeding and thromboembolic events in this unique population. Our data reveal that patients on LD dabigatran were significantly older, had much higher CHADS2 and CHA2DS2-VASC scores, and higher prevalence of diabetes than those from the warfarin arm of the RE-LY trial. In addition, only a minority of these patients qualified for LD dabigatran based on their CrCl (<18%) and none qualified for the LD dabigatran based on drug–drug interactions. This further strengthens the hypothesis that other factors such as advanced age, less severe kidney dysfunction, and other comorbidities play a role in dabigatran dosing with some health-care providers data on clinical outcomes such as bleeding and thromboembolic events from the low-dose dabigatran population are too few to make any conclusive comparisons and are presented in this study as exploratory data. Of note, the most common source of bleeding was gastrointestinal and of all bleeding events, only two, both with gastrointestinal bleeding required red blood cell transfusion.

With an aging population, the prevalence of AF will continue to rise. Such patients will have increasing number of comorbidities that will also increase the incidence of both thromboembolic and bleeding events. Clinical understanding of the safety and efficacy of any agent used in such population is crucial. LD dabigatran, while approved for use in patients with severe renal dysfunction, appears to be used primarily in older patients with multiple comorbidities and not necessarily those with renal dysfunction or drug–drug interactions. This study is the first of its kind to present patient characteristics as well as exploratory clinical outcomes in patients with LD dabigatran. It is important to note that this study has many limitations. These include the retrospective nature of the study that predisposes it to multiple limitations including selection bias. Furthermore, our study population was relatively small thus limiting the power of the analyses. Finally, our study population included only male veterans from a single medical center and thus the results may not be applicable to women or general population. Considering the increasing prevalence of NVAF in the aging US population and the increasing use of DOACs in such patients, further clinical data in the form of larger real world experience or prospective analyses are warranted to better understand the role of LD dabigatran in patients with NVAF.

## Ethics Statement

This study was carried out in accordance with the recommendations of Veterans Affairs Greater Los Angeles Healthcare System Research & Development Committee (IRB exempt). This study was approved with a waiver of written informed consent for the entire study.

## Author Contributions

Research and development preparation and submission: RE and MT. Data collection: SG and MT. Data analysis: RE, JH, and ZF. Manuscript preparation: RE, JH, SG, MT, and ZF.

## Conflict of Interest Statement

The authors declare that the research was conducted in the absence of any commercial or financial relationships that could be construed as a potential conflict of interest. The reviewer CK and handling editor declared their shared affiliation, and the handling editor states that the process nevertheless met the standards of a fair and objective review.
